# Supplementary figures and images for: The suitability of native flowers as pollen sources for Chrysoperla lucasina (Neuroptera: Chrysopidae)
Source: PLoS One. 2020 Oct 23;15(10):e0239847. doi: 10.1371/journal.pone.0239847 (PMC7584243; doi:10.1371/journal.pone.0239847)

A)

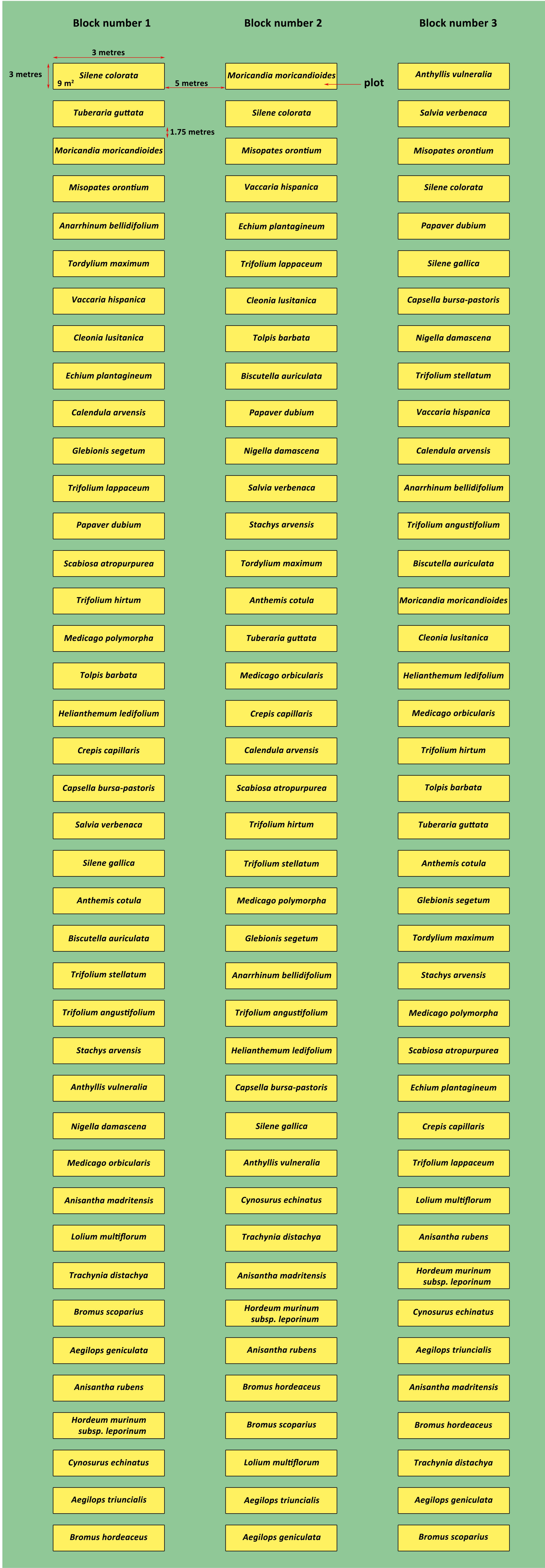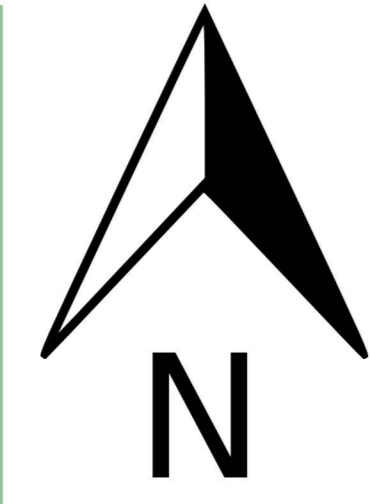

2016

2017

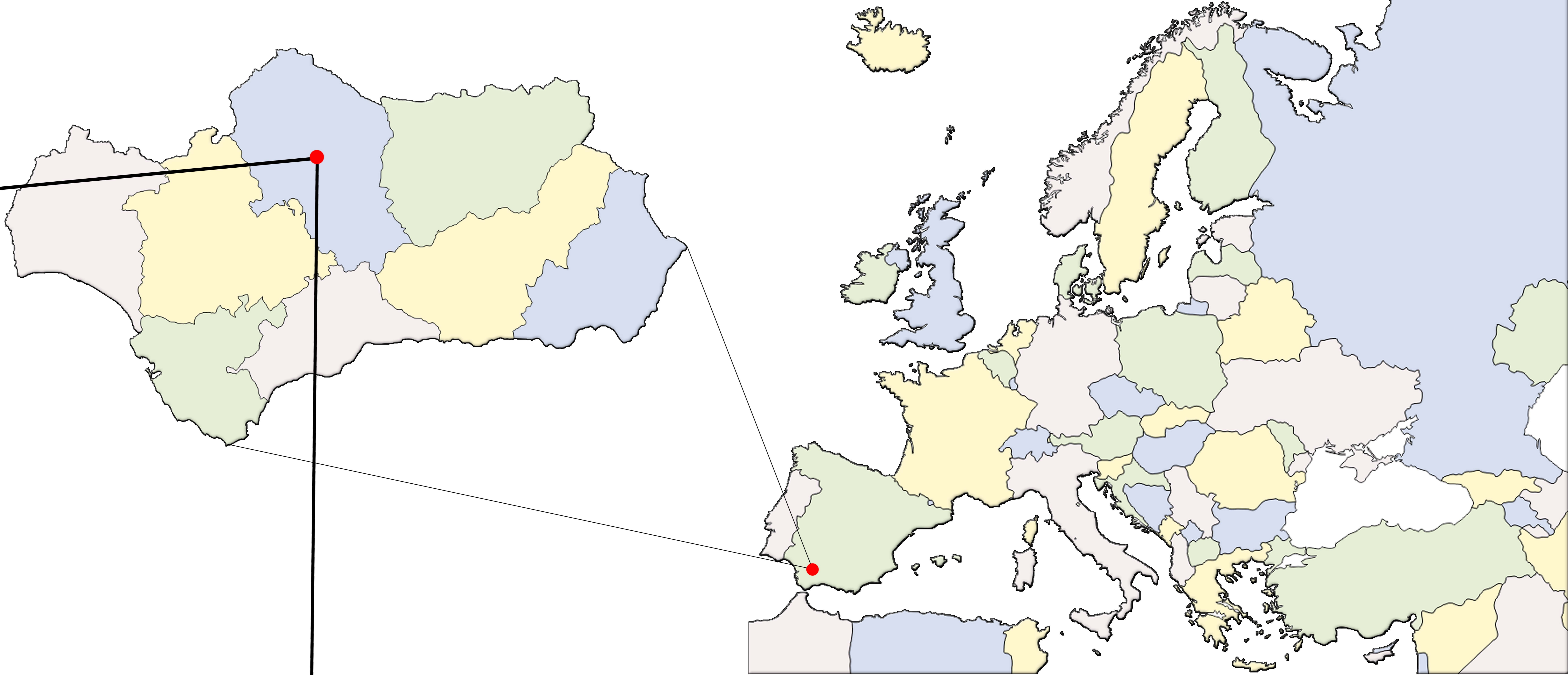

B)

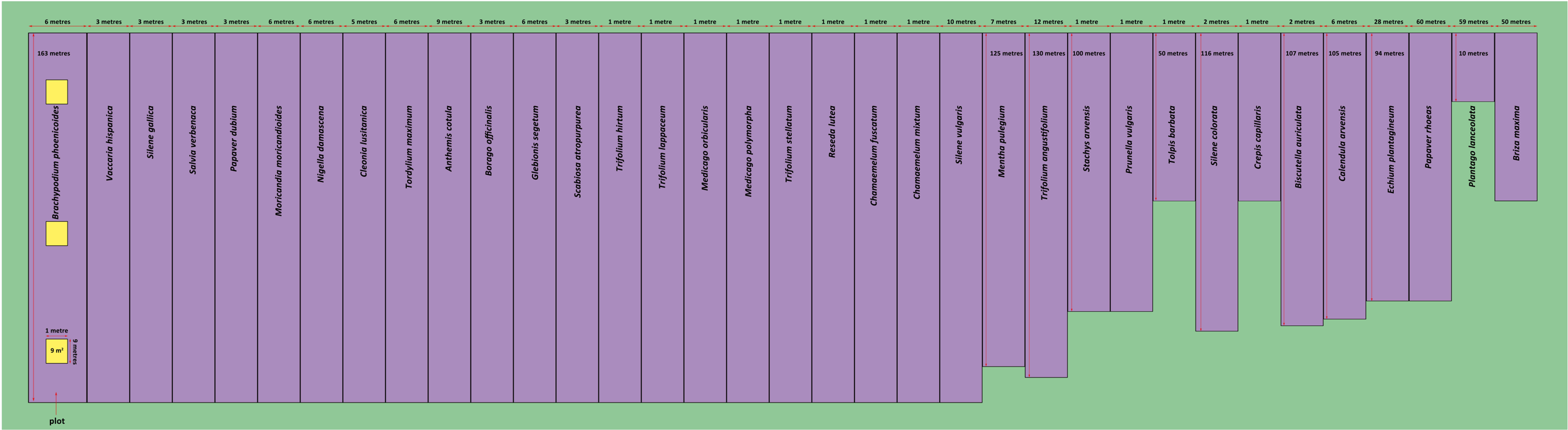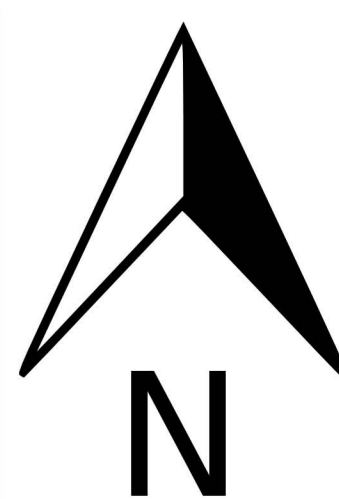

S2 Fig. Field layout with sown plant species distribution in the experimental farm in 2016 (A) and 2017 (B).

Supplement: S1 Fig — Field layout with sown plant species distribution in the experimental farm in 2016 (A) and 2017 (B). (PDF) [file pone.0239847.s001.pdf]
