# Supplementary material for: The suitability of native flowers as pollen sources for Chrysoperla lucasina (Neuroptera: Chrysopidae)
Source: PLoS One. 2020 Oct 23;15(10):e0239847. doi: 10.1371/journal.pone.0239847 (PMC7584243; doi:10.1371/journal.pone.0239847)
Supplement: S2 Fig — Biscutella auriculata (A and B) and Capsella bursa-pastoris (C and D) flowers; photomicrographs of B. auriculata (E) and C. bursa-pastoris (F) pollen grains taken by a confocal microscope at 1000x magnification. Both images (E and F) show exine autofluorescence after merging multiple optical sections; microscopic images of B. auriculata (G) and C. bursa-pastoris (H) pollen grains at 1000x magnification. (PDF) [file pone.0239847.s002.pdf]

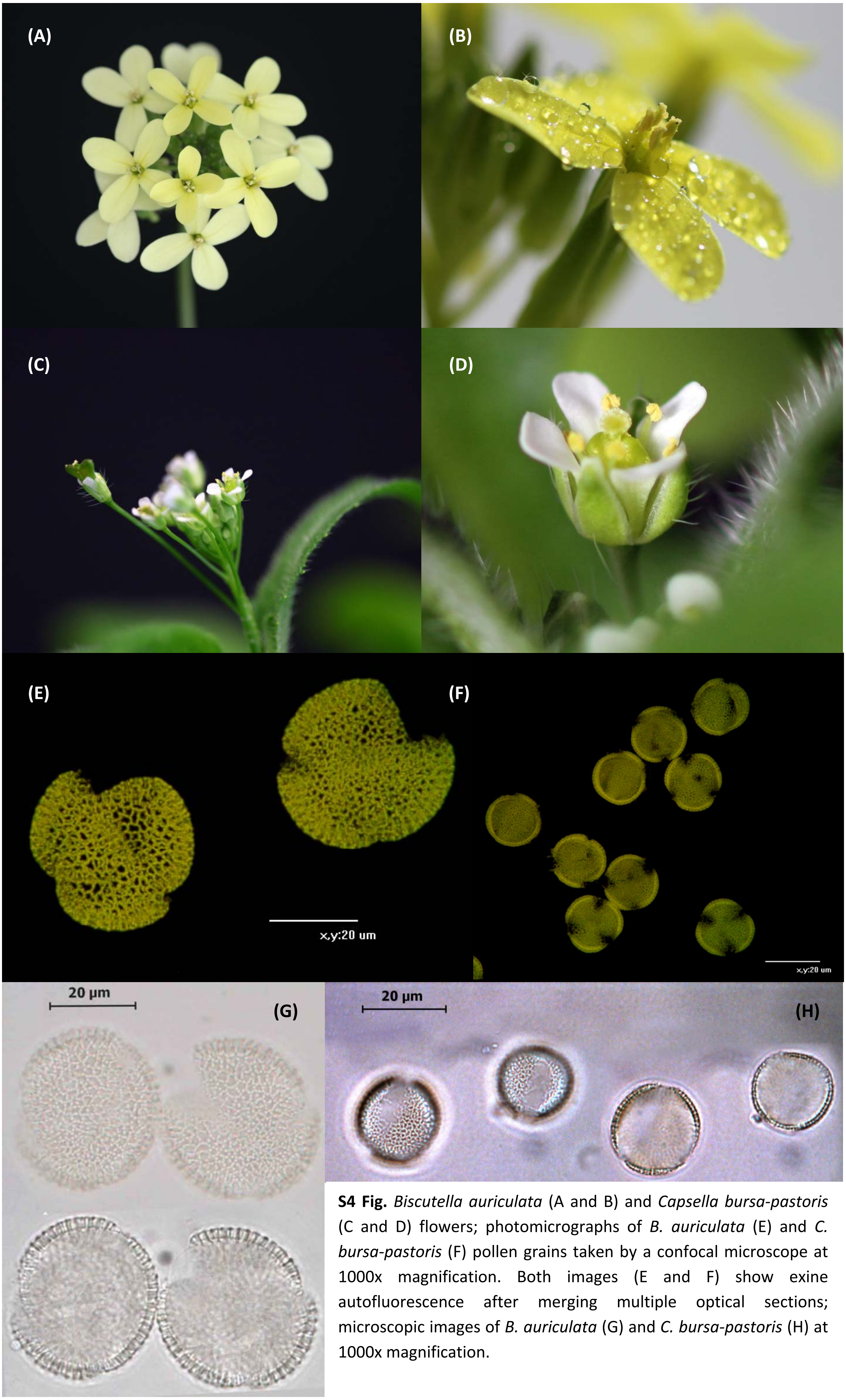

S4 Fig. *Biscutella auriculata* (A and B) and *Capsella bursa-pastoris* (C and D) flowers; photomicrographs of *B. auriculata* (E) and *C. bursa-pastoris* (F) pollen grains taken by a confocal microscope at 1000x magnification. Both images (E and F) show exine autofluorescence after merging multiple optical sections; microscopic images of *B. auriculata* (G) and *C. bursa-pastoris* (H) at 1000x magnification.
