# Supplementary material for: The suitability of native flowers as pollen sources for Chrysoperla lucasina (Neuroptera: Chrysopidae)
Source: PLoS One. 2020 Oct 23;15(10):e0239847. doi: 10.1371/journal.pone.0239847 (PMC7584243; doi:10.1371/journal.pone.0239847)
Supplement: S1 Table — (PDF) [file pone.0239847.s003.pdf]

S1 Table. List of plant species used in the study.

Family	Species
Apiaceae	*Tordylium maximum* L.
Asteraceae	*Anthemis cotula* L.
	Chamaemelum fuscatum (Brot.) Vasc.
	Chamaemelum mixtum (L.) All.
	Glebionis segetum L.
	Calendula arvensis M.Bieb
	Crepis capillaris (L.) Wall.
	Tolpis barbata (L.) Gaertn.
Boraginaceae	*Borago officinalis* L.
	Echium plantagineum L.
Brassicaceae	*Biscutella auriculata* L.
	Capsella bursa-pastoris (L.) Medik.
	Moricandia moricandioides Boiss.
Caprifoliaceae	*Scabiosa atropurpurea* L.
Caryophyllaceae	*Silene gallica* L.
	Silene colorata Poir.
	Silene vulgaris (Moench) Garcke
	Vaccaria hispanica (Mill.) Rauschert
Cistaceae	*Helianthemum ledifolium* (L.) Mill.
	Tuberaria guttata (L.) Fourr.
Fabaceae	*Trifolium hirtum* All.
	Anthyllis vulneraria L.
	Trifolium angustifolium L.
	Trifolium lappaceum L.
	Trifolium stellatum L.
	Medicago orbicularis (L.) Bartal.
	Medicago polymorpha L.
Lamiaceae	*Stachys arvensis* L.
	Cleonia lusitanica L.
	Mentha pulegium L.
	Prunella vulgaris L.
	Salvia verbenaca L.
Papaveraceae	*Papaver dubium* L.
	Papaver rhoeas L.
Plantaginaceae	*Anarrhinum bellidifolium* (L.) Willd.
	Misopates orontium (L.) Raf.
	Plantago lanceolata L.
Poaceae	*Briza maxima* L.
	Cynosurus echinatus L.
	Lolium multiflorum Lam.
	Aegilops geniculata Roth.

Continued S1 Table

Poaceae	*Aegilops triuncialis* L.
	Anisantha madritensis L.
	Anisantha rubens L.
	Brachypodium phoenicoides (L.) Roem. & Schult.
	Bromus hordeaceus L.
	Bromus scoparius L.
	Hordeum murinum subsp. *leporinum* L.
	Trachynia distachya (L.) Beauv.
Ranunculaceae	*Nigella damascena* L.
Resedaceae	*Reseda lutea* L.
