# Supplementary material for: The suitability of native flowers as pollen sources for Chrysoperla lucasina (Neuroptera: Chrysopidae)
Source: PLoS One. 2020 Oct 23;15(10):e0239847. doi: 10.1371/journal.pone.0239847 (PMC7584243; doi:10.1371/journal.pone.0239847)
Supplement: S2 Table — Y–Yes, N–No. (PDF) [file pone.0239847.s004.pdf]

S3 Table. Complete list of plant families, pollen types, plant species, year sown, year sampled, chrysopid collected, bloom period, plant type and lacewing feeding studies references. Y – Yes, N – No.

Family	Pollen type	Plant species	Sown 2016/2017	Sampled 2016/2017	Chrysopids collected 2016/2017	Bloom period	Plant type	Cited in lacewing feeding studies
Apiaceae	*Type Apiaceae	**Foeniculum vulgare* Mill.	N/N	N/N	N/N	V-IX	forb	[1–5]
	Type *Orlaya daucoides*	*Tordylium maximum* L.	Y/Y	Y/N	Y/N	V-VI	forb	
Asteraceae	Type *Anthemis arvensis*	*Anthemis cotula* L.	Y/Y	Y/Y	N/Y	IV-VI	forb	[3,5]
		Chamaemelum fuscatum (Brot.) Vasc.	N/Y	N/N	N/N	X-V	forb	
		Chamaemelum mixtum (L.) All.	N/Y	N/N	N/N	II-XI	forb	
		Glebionis segetum L.	Y/Y	Y/Y	Y/Y	II-VI	forb	[1,5]
	Type *Calendula arvensis*	*Calendula arvensis* M.Bieb	Y/Y	Y/Y	N/N	II-X	forb	[2]
	Type *Crepis capillaris*	*Crepis capillaris* (L.) Wall.	Y/Y	Y/Y	Y/Y	III-IX	forb	[1]
		Tolpis barbata (L.) Gaertn.	Y/Y	Y/Y	N/Y	IV-VIII	forb	
Boraginaceae	Type *Borago officinalis*	*Borago officinalis* L.	N/Y	N/Y	N/Y	II-VI	forb	[3]
	Type *Echium plantagineum*	*Echium plantagineum* L.	Y/Y	Y/Y	Y/Y	II-VI	forb	[1,3,5]
Brassicaceae	Type *Capsella bursa-pastoris*	*Biscutella auriculata* L.	Y/Y	Y/Y	Y/Y	II-VI	forb	[3,5]
		Capsella bursa-pastoris (L.) Medik.	Y/N	Y/N	N/N	XII-V	forb	[1,3,5]
	Type *Raphanus raphanistrum*	*Moricandia moricandioides* Boiss.	Y/Y	Y/Y	Y/Y	III-VI	forb	
Caprifoliaceae	Type *Scabiosa atropurpurea*	*Scabiosa atropurpurea* L.	Y/Y	Y/Y	Y/Y	I-XII	forb	
Caryophyllaceae	Type *Silene latifolia*	*Silene gallica* L.	Y/Y	Y/Y	N/Y	II-XI	forb	[5]
	Type *Silene vulgaris*	*Silene colorata* Poir.	Y/Y	Y/Y	Y/Y	I-VI	forb	[5]
		Silene vulgaris (Moench) Garcke	N/Y	N/N	N/N	II-XI	forb	[5]
	Type *Vaccaria hispanica*	*Vaccaria hispanica* (Mill.) Rauschert	Y/Y	Y/Y	Y/N	IV-VI	forb	[1]
Cistaceae	Type *Helianthemum ledifolium*	*Helianthemum ledifolium* (L.) Mill.	Y/N	N/N	N/N	II-VI	forb	[5]
	Type *Tuberaria guttata*	*Tuberaria guttata* (L.) Fourr.	Y/N	Y/N	N/N	II-VII	forb	

Continued S3 Table

Ericaceae	*Type Ericaceae	**Erica* sp.	N/N	N/N	N/N	XII-VI	forb	[1,3]
Fabaceae	Type Fabaceae	*Trifolium hirtum* All.	Y/Y	Y/Y	N/Y	V-VI	forb	[5]
	Type *Lotus creticus*	*Anthyllis vulneraria* L.	Y/N	Y/N	N/N	VI-IX	forb	
	Type *Trifolium arvense*	*Trifolium angustifolium* L.	Y/Y	Y/Y	Y/Y	III-VIII	forb	
		Trifolium lappaceum L.	Y/Y	Y/Y	N/Y	IV-VII	forb	
		Trifolium stellatum L.	Y/Y	Y/N	Y/N	III-VI	forb	
		**Vicia villosa* Roth.	N/N	N/N	N/N	III-VII	forb	[5]
	Type *Trifolium repens*	*Medicago orbicularis* (L.) Bartal.	Y/Y	Y/N	Y/N	IV-VI	forb	[1]
		Medicago polymorpha L.	Y/Y	Y/N	N/N	III-VII	forb	[1]
Fagaceae	*Type *Castanea sativa*	**Castanea sativa* Mill.	N/N	N/N	N/N	V-VII	tree	[5]
Lamiaceae	Type *Lamium amplexicaule*	*Stachys arvensis* L.	Y/Y	Y/Y	N/Y	II-V	forb	[5,6]
	Type *Mentha aquatica*	*Cleonia lusitanica* L.	Y/Y	Y/N	Y/N	IV-VII	forb	
		Mentha pulegium L.	N/Y	N/N	N/N	V-X	forb	[5]
	Type *Salvia verbenaca*	*Prunella vulgaris* L.	N/Y	N/N	N/N	V-VIII	forb	[5]
		Salvia verbenaca L.	Y/Y	Y/N	N/N	XI-VI	forb	[5]
Papaveraceae	Type *Papaver rhoeas*	*Papaver dubium* L.	Y/Y	Y/Y	N/Y	III-VI	forb	[5]
		Papaver rhoeas L.	Y/Y	Y/Y	N/Y	II-VI	forb	[1,5]
Pinaceae	*Type *Pinus pinea*	**Pinus* sp.	N/N	N/N	N/N	III-V	tree	[1,3,5]
Plantaginaceae	Type *Anarrhinum bellidifolium*	*Anarrhinum bellidifolium* (L.) Willd.	Y/N	N/N	N/N	IV-VI	forb	
	Type Plantaginaceae	*Misopates orontium* (L.) Raf.	Y/N	Y/N	N/N	III-VI	forb	[3]
	Type *Plantago coronopus*	*Plantago lanceolata* L.	N/Y	N/N	N/N	IV-VI	forb	[1,3,5,6]
Poaceae	Type *Festuca arundinacea*	*Briza maxima* L.	N/Y	N/N	N/N	III-VII	grass	
		Cynosurus echinatus L.	Y/N	Y/N	Y/N	IV-VII	grass	
		Lolium multiflorum Lam.	Y/N	Y/N	Y/N	IV-VI	grass	

Continued S3 Table

Poaceae	Type Poaceae	*Aegilops geniculata* Roth.	Y/N	N/N	N/N	IV-VII	grass	[1,3,5]
		Aegilops triuncialis L.	Y/N	N/N	N/N	V-VII	grass	
		Anisantha madritensis L.	Y/N	Y/N	N/N	III-V	grass	
		Anisantha rubens L.	Y/N	Y/N	N/N	IV-V	grass	
		Brachypodium phoenicoides (L.) Roem. & Schult.	N/Y	N/N	N/N	VI-VII	grass	
		Bromus hordeaceus L.	Y/N	Y/N	Y/N	III-VI	grass	
		Bromus scoparius L.	Y/N	Y/N	N/N	III-V	grass	
		Hordeum murinum subsp. *leporinum* L.	Y/N	Y/N	Y/N	II-V	grass	
		Trachynia distachya (L.) Beauv.	Y/N	Y/N	Y/N	III-VI	grass	
Ranunculaceae	Type *Nigella damascena*	*Nigella damascena* L.	Y/Y	Y/Y	Y/Y	III-VI	forb	
Resedaceae	Type *Reseda luteola*	*Reseda lutea* L.	N/Y	N/N	N/N	III-VII	forb	
Rhamnaceae	*Type Rhamnaceae	**Rhamnus* sp.	N/N	N/N	N/N	I-VII	forb	[5]

*Pollen type identified from surrounding vegetation.

References

1. Villenave-Chasset J, Denis A. Study of pollen consumed by Lacewings (Neuroptera, Chrysopidae) and Hoverflies (Diptera, Syrphidae) in Western France. Symbioses. 2013;29: 17–20.

2. Villa M, Santos SAP, Benhadi-Marín J, Mexia A, Bento A, Pereira JA. Life-history parameters of Chrysoperla carnea s.l. fed on spontaneous plant species and insect honeydews: importance for conservation biological control. BioControl. 2016;61: 533–543. doi:10.1007/s10526-016-9735-2

3. Villa M, Somavilla I, Santos SAP, López-Sáez JA, Pereira JA. Pollen feeding habits of Chrysoperla carnea s.l. adults in the olive grove agroecosystem. Agric Ecosyst Environ. 2019;283: 106573. doi:https://doi.org/10.1016/j.agee.2019.106573

4. Resende ALS, Souza B, Ferreira RB, Aguiar-Menezes EL. Flowers of Apiaceous species as sources of pollen for adults of Chrysoperla externa (Hagen) (Neuroptera). Biol Control. 2017;106: 40–44. doi:10.1016/j.biocontrol.2016.12.007

5. Bertrand C, Eckerter PW, Ammann L, Entling MH, Gobet E, Herzog F, et al. Seasonal shifts and complementary use of pollen sources by two bees, a

lacewing and a ladybeetle species in European agricultural landscapes. J Appl Ecol. 2019;56: 2431–2442. doi:10.1111/1365-2664.13483

6. Nunes Morgado L, Resendes R, Moura M, Mateus Ventura MA. Pollen resources used by Chrysoperla agilis (Neuroptera: Chrysopidae) in the Azores, Portugal. Eur J Entomol. 2014;111: 143–146.
