# Supplementary material for: The suitability of native flowers as pollen sources for Chrysoperla lucasina (Neuroptera: Chrysopidae)
Source: PLoS One. 2020 Oct 23;15(10):e0239847. doi: 10.1371/journal.pone.0239847 (PMC7584243; doi:10.1371/journal.pone.0239847)
Supplement: Attachment — Submitted filename: Response to Reviewers.docx [file pone.0239847.s005.docx]

Reviewers' comments:

Reviewer's Responses to Questions

**Comments to the Author**

1. Is the manuscript technically sound, and do the data support the conclusions?

The manuscript must describe a technically sound piece of scientific research with data that supports the conclusions. Experiments must have been conducted rigorously, with appropriate controls, replication, and sample sizes. The conclusions must be drawn appropriately based on the data presented.

Reviewer #1: Partly

Reviewer #2: Yes

**Response: We have modified the text according to both reviewers’ comments to submit a well written technically sound manuscript and to ensure that the data support the conclusions.**

2. Has the statistical analysis been performed appropriately and rigorously?

Reviewer #1: N/A

Reviewer #2: Yes

**Response: The statistical analysis has been carried out in accordance with the reviewers’ comments.**

3. Have the authors made all data underlying the findings in their manuscript fully available?

The [PLOS Data policy](http://www.plosone.org/static/policies.action#sharing) requires authors to make all data underlying the findings described in their manuscript fully available without restriction, with rare exception (please refer to the Data Availability Statement in the manuscript PDF file). The data should be provided as part of the manuscript or its supporting information, or deposited to a public repository. For example, in addition to summary statistics, the data points behind means, medians and variance measures should be available. If there are restrictions on publicly sharing data—e.g. participant privacy or use of data from a third party—those must be specified.

Reviewer #1: No

Reviewer #2: Yes

**Response: We have modified the material and methods and results sections in order to highlight our findings. We also promise to provide the DIGITAL CSIC repository information when the article is accepted for publication.**

4. Is the manuscript presented in an intelligible fashion and written in standard English?

PLOS ONE does not copyedit accepted manuscripts, so the language in submitted articles must be clear, correct, and unambiguous. Any typographical or grammatical errors should be corrected at revision, so please note any specific errors here.

Reviewer #1: Yes

Reviewer #2: Yes

**Response: Thank you for your comments. We have modified the English according to your comments and a native English speaker has corrected the text prior to submission of the revised manuscript.**

5. Review Comments to the Author

Please use the space provided to explain your answers to the questions above. You may also include additional comments for the author, including concerns about dual publication, research ethics, or publication ethics. (Please upload your review as an attachment if it exceeds 20,000 characters)

**Reviewer #1:** In the current manuscript, the authors investigated the feeding behaviour of the chrysopid species *Chrysoperla lucasina* for different types of pollen obtained from 51 native plant species from an experimental farm in Spain. The authors found that this chrysopid species fed on various plants, consuming 17 out of the 29 pollen types available. Some methods and results presented by the authors are not reliable and some details are missing. In the method section, the sampling area and the plant species used by the authors are not mentioned. They also don’t mention the number of plants used (replicates) and how plants were distributed in the farm. Something that also needs be noted is the number of insects that the authors sampled. In 2016, they sampled 45 individuals and in 2017, they sampled 62. If the authors sown 51 plant species in the farm, sampling only 45 chrysopid is not enough. Furthermore, in several parts of the manuscript, the authors claimed they have tested the feeding preference of the chrysopid, which is also misleading. They did not perform any preference test. Table and figures need to be improved (need to be more informative and should align with the goals of the manuscript). Therefore, due to all these issues, I am rejecting the publication of this manuscript.

I recommend the authors to be focused on their goals and on the scientific contribution of the manuscript while re-writing the text and I hope my comments bellow help with that.

**Response: Thank you for all your comments and for taking the time to review the manuscript. We have taken all your comments into consideration and have substantially modified the introduction, material and methods, results, discussion and conclusion sections accordingly in order to focus more clearly on our goals. We have added more detail in the material and methods section and have modified the tables and figures. All chrysopid adults collected in 2016 (63 individuals) and 2017 (46 individuals) were used to investigate the pollen types consumed on the 29 pollen types available belong to 51 native plant species sown. If we have collected more chrysopid adults, we have used more to investigate the pollen types consumed.**

**COMMENTS TO THE AUTHORS:**

Abstract: Line 14: Species name should be writing without abbreviation when cited for the first time.

**Response: We have modified the text (line 15).**

Line 24: Can you be sure that the insects consumed honeydew?.

**Response: The presence of fungal spores in the canal alimentary is an indirect indicator that chrysopids consume honeydew on plant surfaces, which is in line with the findings of Villenave doctoral thesis (2006) and Villenave et al. (2006). Villa et al. (2016) also show how *C. carnea s.l.* feed on insect honeydew.**

**Villenave J. Etude de la Bio-écologie des Névropteres dans une perspective de lutte biologique par conservation. Université d’Angers. 2006.**

**Villenave J, Deutsch B, Lode T, Rat-Morris E. Pollen preference of the Chrysoperla species (Neuroptera: Chrysopidae) occurring in the crop environment in western France. Eur J Entomol. 2006;103: 771–777.**

**Villa M, Santos SAP, Benhadi-Marín J, Mexia A, Bento A, Pereira JA. Life-history parameters of Chrysoperla carnea s.l. fed on spontaneous plant species and insect honeydews: importance for conservation biological control. BioControl. 2016;61: 533–543. doi:10.1007/s10526-016-9735-2**

**Introduction:**

The manuscript is related to native plants of a specific region in South of Spain, but in the introduction, the authors don’t provide info for questions such as: Why is it important to study this region? Why is it important to study green lacewings (more specific, *C. lucasina*) in this area? What is the problem you can try to solve? I suggest to author include some info about this. Write more clearly the aims of the study and follow it through the text.

**Response: We have modified the text to create a clearer structure for the main areas covered in your questions (lines 33 to 124).**

Line 36: I suggest:"…to minimize negative effects in the environment through ..."

**Response: Thank you for your comment. We have not modified the text on the advice of our proofreader (line 41).**

Line 40: What are semi-natural habitats (SNHs)? Maybe include just one sentence to explain it better, because right after that, you talk about “low SNHs.

**Response: We have modified the text (lines 43-46).**

Line 45: I suggest “Flowering plants attracts insects through its floral resources, ...” **Response: We have modified the text (lines 54-55).**

Line 47: When the author says entomophagous insects, I suggest to add some explanation or synonym such as predator, carnivorous insects or natural enemies.

**Response: We have modified the text (line 56).**

Line 51: The conclusion of the sentence (“depending on the target arthropod”) seems loose.

**Response: We have modified the text and deleted “depending on the target arthropod” (lines 64-65).**

Line 54: Is it correct to say “complex *Chrysoperla carnea* (Stephens)”?

**Response: Yes, it is. The species *Chrysoperla carnea* (Stephens) contains at least 21 sibling species named complex *Chrysoperla carnea* (Stephens) or *Chrysoperla carnea sensu lato* (Stephens) (Henry et. al 2002, Henry et. al 2013 and Chapman et. al 2006).**

**Henry CS, Brooks SJ, Duelli P, Johnson JB. Discovering the true Chrysoperla carnea (Insecta: Neuroptera: Chrysopidae) using song analysis, morphology, and ecology. Ann Entomol Soc Am. 2002;95: 172–191. doi:10.1603/0013-8746(2002)095[0172:dttcci]2.0.co;2**

**Henry CS, Brooks SJ, Duelli P, Johnson JB, Wells MM, Mochizuki A. Obligatory duetting behaviour in the Chrysoperla carnea-group of cryptic species (Neuroptera: Chrysopidae): its role in shaping evolutionary history. Biol Rev. 2013;88: 787–808. doi:10.1111/brv.12027**

**Chapman JW, Reynolds DR, Brooks SJ, Smith AD, Woiwod IP. Seasonal variation in the migration strategies of the green lacewing Chrysoperla carnea species complex. Ecol Entomol. 2006;31: 378–388. doi:10.1111/j.1365-2311.2006.00797.x**

Line 64: I suggest you to remove this sentence (*C. carnea* require a floral architecture sufficiently large for their thorax and mouthpart to reach the pollen and well-exposed nectaries to gain access to nectar [27-29].).

**Response: We have deleted the text according to your comment (lines 83-84).**

Line 76: “species-richness inventories” are referring to plant species or insect species?

**Response: It is referred to plant species. We have modified the text (lines 96-97).**

This paragraph needs a conclusion sentence.

**Response: We have added a conclusion sentence (lines 106-108).**

**Material and Methods**

I suggest you to split the topic “Study area” in two: “Study area” and “Insect collection” (or something like that). Again, why is it important to study green lacewings in this area? What is the problem to be solved? In the topic “Insect collection” write more details about the sampling effort, how the sampling was made….

Include details about the native species that were sown and why they were selected?

**Response: We have split the section into two separate subsections entitled “Study area” and “Insect collection”. We have added details and have included a reference to another study of ours which used the same experimental farm and insect sampling approach (lines 126-168).**

**Results**

The results are confused, as well as the Table 1.

**Response: Thank you for your comment. We have modified the text to create a clearer structure for the results section, tables and figures (lines 232-334). We have also divided the table 1 into three tables and one figure (revised Table 1, S1 Table, S2 Figure and S3 Table).**

- **Table 1 shows the results for the pollen types identified.**
- **S1 Table contains a list of plant species used.**
- **S2 Fig shows a field layout with the sown plant species distribution.**
- **S3 Table contains detailed information on all the plant species, seeds, samples and chrysopids collected. Additional information is also provided on the pollen and plant types belonging to the plant species, blooming periods and previous studies of lacewing feeding behavior.**

The figure 1 have two variables mixed: pollen type and plant species, is it correct?

**Response: Yes, it is. Our primary aim was to match *B. auriculata* and *C. bursa-pastoris* plant species with different pollen types. Figure 1, which has been changed to Figure 2, now only shows pollen types.**

Line 179 to 185: In the first part, you used “first, second and third” to refer to groups. Then, you use numbers “1, 2, 3 and 4”. I suggest you to choose only one way to refer to groups.

**Response: We have modified the text (lines 302-314).**

**Discussion**

In general, I suggest you to explore more your results.

**Response: We have modified the text to explore the results more thoroughly (lines 335-447).**

Lines 224 to 232: this paragraph could be in the result section. It is simple and clear.

**Response: We have moved part of this paragraph to the results section (lines 254-263).**

**Conclusion**

Line 272: “We specifically suggest that the sources of pollen include the following native plant species … which are the most frequently consumed by *C. lucasina*.” Do you suggest the use of these species everywhere or only at your study region? Be more cautious with your conclusions and keep the focus.

**Response: We have modified the text (lines 449-463).**

**Reviewer #2:** I have reviewed the article entitled “The suitability of native flowers as pollen source for *Chrysoperla lucasina* (Neuroptera: Chrysopidae)”. This is an interesting article addressing an important topic for conservation biological control of pests. Despite that there are other similar studies published, I believe it is important that this study is focused on *C. lucasina*, which was previously neglected or confounded with *C. carnea*, so any advance on knowing the feeding behavior and ecology of the species from the carnea complex will help us understand more about their ecology, the differences between and, from an applied point of view, how to enhance their abundance in cultivated fields.

I congratulate the authors for their work, given that identifying the pollen grains is complicated, and the use of the transparency method seems like a good option, easier than the acetolysis process that is more commonly used. Nevertheless, I think that the article would benefit from some changes before being published. Below, I provide my main concerns and some minor suggestions to the authors.

**Response: Thank you for your comments and for taking the time to review the manuscript. We have modified the manuscript according to your suggestions and hope that it has been sufficiently improved to be published.**

**Major comments:**

-The introduction is good and deals with relevant topics for the study system. However, at some points, it can be improved. The first paragraph would benefit from more connectivity between the sentences, as some of them are a bit disconnected, leading to the ecological intensification of agriculture. At the end of the second paragraph, the authors jump directly to chrysopids when they were talking about biological control and natural enemies in general. I believe that the link with lacewings can be included directly in the next paragraph or that the last sentence should be slightly modified to incorporate them in this general context. The third paragraph is a bit disorganized and would benefit from a better structure when talking about different aspects of lacewings biology and feeding preferences.

**Response: We have modified the first paragraph of the introduction by enhancing the linkages (lines 34 to 46). In the second paragraph, we have added a sentence which connects with the third paragraph (lines 61 to 65). We have also reorganized the third and fourth paragraphs (lines 66 to 108).**

-The objectives of the article focus on pollen feeding preferences but do not mention the differences between sexes and the importance of honeydew for lacewings. However, both topics are addressed in the results and specific figures are shown. I think both topics are relevant, especially honeydew consumption, so the authors could include these as specific objectives or questions. During the introduction and methods, the fact that some lacewings feed on honeydew in briefly mentioned, but only at the end of the discussion the authors explain that this can be identified by the presence of fungal spores. I think this should be clarified before, in the introduction or methods section.

**Response: We have clarified the objectives (lines 109 to 124) and have included additional information regarding honeydew in the material and methods section (lines 198 to 199).**

-The plant species sown are listed in table 1 in the results section and, in methods, this is only briefly mentioned (L. 98). However, the selection and sowing of the species is part of their methods, so I think that the reasons for their choice can be included before. The authors could refer to table 1 in methods, but explain if the plant species included flowers with different traits, flowering periods, previous reports on lacewing feeding, etc.

**Response: Thank you for your comment. We have added more information in the material and methods section on the plant selection process (lines 137 to 144). We have also divided Table 1 into three distinct tables and one figure (revised Table 1, S1Table, S2 Fig and S3 Table).**

- **Table 1 shows the results for the pollen types identified.**
- **S1 Table contains a list of the plant species used.**
- **S2 Fig shows a field layout with the spatial distribution of the sown plants.**
- **S3 Table contains detailed information on the plant species, seeds, samples and number of chrysopids collected. We also included information on pollen types and plant species families, blooming periods and previous studies of lacewing feeding behavior.**

-In the multivariate analyses of pollen consumption, the authors refer to the overlap between diets in results, but this could be explored explicitly. A specific test such as an ANOSIM or similar could be done to determine if the differences between sexes on the diet composition were significantly different or not.

**Response: Although we have carried out PERMANOVA and ANOSIM analyses, which produced similar results, we opted for PERMANOVA which is generally regarded as more robust. We performed an additional PERMANOVA analysis to determine any differences in dietary composition between the sexes. We have modified the statistical analysis and results sections (lines 213-231).**

-As with the objectives section, I think the results section could be organized to refer to different specific objectives. The section has some order, but I have the impression that results from different questions are mixed and can be organized better. In my opinion, different parts that can be separated better are the description of the diet including the presence of surrounding plants, feeding preferences (including the multivariate analyses), differences by sex, presence of spores, and honeydew.

**Response: We have improved the structure of the results section in line with the specific objectives of the study (lines 232-334).**

-In the discussion (l. 226-231) and conclusions (l. 272-275) the mention of a lot of plant species names becomes too repetitive. Furthermore, the temporal variation in the diet of lacewings should be discussed more, as the authors only sampled in May, and the diet changes through time. Regarding this, a recent and very relevant paper (Bertrand et al. 2019, J Appl Ecol, 56(11), 2431-2442) should be also mentioned.

**Response: Thank you for your comment. We have modified the text and have added a reference to the study by Bertrand (335-447), which unfortunately we discovered shortly after submitting the article to the PLOS ONE system.**

-The article is, in general, well written, but would benefit from the language edit by a native English speaker. The parts where corrections are necessary are just a few, but it will improve the text flow. Some of these details are highlighted below in my minor comments.

**Response: Thank you for your comments on the quality of the English in the manuscript, which was corrected by a native English speaker before being submitted to PLOS ONE the first time. The article has now been revised by a professional proofreader.**

**Minor comments:**

-The title should say "as pollen sources" or "as a pollen source" instead.

**Response: We have modified the text (line 1).**

-L. 23: the sentence from point (iii) of the results section of the abstract is not so clearly written. I believe it would be more correct to say that the majority of the pollen types identified correspond to the sown native plants, and no that the pollen was found on the plant species.

**Response: Thank you for your comment. We have modified the text on the advice of our proofreader (line 24-26).**

-Throughout the manuscript, the authors refer to semi-natural habitats as SNH or SNHs, not always because they refer to singular or plural mentions of these habitats. I believe that SNH is widely used in the literature with no need to add the ‘s’ in the end and the same criteria should be applied across the text.

**Response: We have modified this throughout the manuscript.**

-L. 52: the family of natural enemies sounds strange, as it can be confounded with taxonomical families. The group or functional group might be more appropriate.

**Response: We have modified the text (line 66).**

-L. 83: The sentence would be improved by adding “to identify which/the sown native species”.

**Response: We have clarified the objectives (lines 109-124).**

-L. 58: Many references are cited like Villenave, Deutsch in this line. Please check the journal guidelines as I believe that in these cases only the first author should be mentioned before the number of the reference.

**Response: We have reinserted the references using the Mendeley software, as our institutional EndNote license had expired. We have made adjustments to the PLOS ONE reference style.**

-L. 90: the section describes the study area but also lacewing samplings. The title could then be modified to make this clear?

**Response: Thank you for your comment. We have divided the section into two separate sections entitled “Study area” and “Insect collection” (lines 126-168). Where possible, we have simplified these sections and have included a reference to our published article which used the same study area and sampling methodology Alcalá Herrera et al. (2019).**

**Alcalá Herrera R, Ruano F, Gálvez Ramírez C, Frischie S, Campos M. Attraction of green lacewings (Neuroptera: Chrysopidae) to native plants used as ground cover in woody Mediterranean agroecosystems. Biol Control. 2019;139. doi:10.1016/j.biocontrol.2019.104066**

-L. 108: is “peeling off” the best term to describe this? Taking off wings, legs, and antennae might be better.

**Response: We have changed “peeling off” to “removing off” on the advice of our proofreader (line 177).**

-L. 116: the division of the alimentary canal in sections is interesting, but it is not clear if the authors did it after identifying the pollen or if the identification was done without contemplating the location of the pollen grains and the division was done just to show the proportions of the locations. A better explanation of the reasons can be helpful here. **Response: We have clarified the text (lines 186-194).**

-L. 137-138: instead of “in addition to”, the authors might refer to “using the packages”. **Response: We have modified the text (lines 214-215).**

-L. 145: I would not say that including the species that were consumed only a few times would cause disturbances in the analysis, but they could be influential. Thus, perhaps “to minimize the influence of species that were only consumed occasionally” or something similar could be a better expression.

**Response: We have modified the text (lines 220-221).**

-Table 1 could be organized in a different way to show the relative importance of plant families. Instead of organizing it alphabetically by pollen type names, can the authors organize it alphabetically by plant family and then by pollen type within each family? **Response: Thank you for your comment. We have reorganized the plant families alphabetically and their corresponding pollen types. We have also split Table 1 into three separate tables and one figure (revised Table 1, S1 Table, S2 Fig and S3 Table).**

- **Table 1 shows the results for the pollen types identified.**
- **S1 Table contains a list of the plant species used.**
- **S2 Fig is a field layout of the sown plant species.**
- **S3 Table provides detailed information on the plant species, seeds, samples and chrysopids collected. We also provide information on pollen types and plant types belonging to the plant species, blooming periods and previous studies of lacewing feeding patterns.**

-L 271-272: pollen sources and resources could be changed to pollen resources or plants providing pollen resources.

**Response: We have modified the text (lines 448-463).**

-Fig. 2 would benefit from titles on each panel for better identification of what it is shown.

**Response: We have modified Fig. 2 which has been retitled Fig 3.**
