# Supplementary material for: The suitability of native flowers as pollen sources for Chrysoperla lucasina (Neuroptera: Chrysopidae)
Source: PLoS One. 2020 Oct 23;15(10):e0239847. doi: 10.1371/journal.pone.0239847 (PMC7584243; doi:10.1371/journal.pone.0239847)
Supplement: Attachment — Submitted filename: Response to reviewers (2nd round).docx [file pone.0239847.s006.docx]

Reviewers' comments:

Reviewer's Responses to Questions

**Comments to the Author**

1. If the authors have adequately addressed your comments raised in a previous round of review and you feel that this manuscript is now acceptable for publication, you may indicate that here to bypass the “Comments to the Author” section, enter your conflict of interest statement in the “Confidential to Editor” section, and submit your "Accept" recommendation.

Reviewer #2: (No Response)

Reviewer #3: All comments have been addressed

2. Is the manuscript technically sound, and do the data support the conclusions?

The manuscript must describe a technically sound piece of scientific research with data that supports the conclusions. Experiments must have been conducted rigorously, with appropriate controls, replication, and sample sizes. The conclusions must be drawn appropriately based on the data presented.

Reviewer #2: Yes

Reviewer #3: Yes

3. Has the statistical analysis been performed appropriately and rigorously?

Reviewer #2: Yes

Reviewer #3: Yes

4. Have the authors made all data underlying the findings in their manuscript fully available?

The [PLOS Data policy](http://www.plosone.org/static/policies.action#sharing) requires authors to make all data underlying the findings described in their manuscript fully available without restriction, with rare exception (please refer to the Data Availability Statement in the manuscript PDF file). The data should be provided as part of the manuscript or its supporting information, or deposited to a public repository. For example, in addition to summary statistics, the data points behind means, medians and variance measures should be available. If there are restrictions on publicly sharing data—e.g. participant privacy or use of data from a third party—those must be specified.

Reviewer #2: Yes

Reviewer #3: Yes

5. Is the manuscript presented in an intelligible fashion and written in standard English? PLOS ONE does not copyedit accepted manuscripts, so the language in submitted articles must be clear, correct, and unambiguous. Any typographical or grammatical errors should be corrected at revision, so please note any specific errors here.

Reviewer #2: Yes

Reviewer #3: Yes

**Response: Thank you for your comments. We have modified the English according to your comments and a native English speaker has corrected the text prior to submission of the revised manuscript.**

6. Review Comments to the Author

Please use the space provided to explain your answers to the questions above. You may also include additional comments for the author, including concerns about dual publication, research ethics, or publication ethics. (Please upload your review as an attachment if it exceeds 20,000 characters)

**Reviewer #2:** In the revised version of the manuscript “The suitability of native flowers as pollen source for *Chrysoperla lucasina* (Neuroptera: Chrysopidae)”, the authors have replied to the comments made by the two reviewers during the first submission. I thank the authors for their work on the revision. They have placed more emphasis on the importance of the study for the Mediterranean region, which helps the reader to understand why this research was relevant. English writing is also improved, but still, some changes are necessary, especially in parts of the discussion. The manuscript is clearer now and the objectives are clearly defined. Nevertheless, I think that the discussion needs more structure and some important topics are omitted. Plus, I found many minor changes that would improve the text. Therefore, I am recommending another major revision.

**Response: Thank you for all your comments and for taking the time to review again the manuscript. We have taken all your comments into consideration and have modified the abstract, introduction, material and methods, results, discussion and conclusion sections accordingly for greater clarity.**

**Major comments:**

-Despite that their previous article (Alcalá Herrera et al. 2019, Biol. Control) is cited in the text, I feel that more links to this paper are necessary, as both works are clearly part of the same project and are highly connected. Particularly, in the discussion, it could be mentioned if the plants that were frequently visited by lacewings in their previous article are the same that were found to be consumed here. By looking at the highlights and abstract, it seems that not all species are shared, at least within the most important. What are the implications of this? Could the recommendations of sown plant species based only on flower visitation be inaccurate?

**Response: Thank you for your comments. We have added more information in the discussion section regarding the previous article (lines 284-434).**

-In general, the discussion structure seems unorganized. The first paragraph is quite long and mixes their main findings with the discussion about other aspects of lacewings’ identification, temporal dynamics, and movement. I suggest to keep the first paragraph short and with a clear mention of the contribution of their study and discuss the other topics in other parts.

**Response: We have modified the text according to your comments (lines 284-434).**

Furthermore, on lines 267-269 the question of whether *C. lucasina* is a generalist or specialist pollen feeder appears, and this was not mentioned before, at least explicitly. The specific objectives could introduce this question more clearly, perhaps.

**Response: We have added a specific objective (lines 104-105) and have modified the text in order to make the discussion section clearer (lines 284-434).**

The paragraph on the competition with other insects such as pollinators on lines 306-313 is disconnected from the rest of the article, as this was not mentioned before, so some changes to improve the connection are needed.

**Response: We have deleted this paragraph (lines 402-409).**

Finally, the result of significant differences between sexes in the percentage of alimentary canal with pollen are not discussed in detail, and this could be important due to the implications of flower resources on reproduction (which is mentioned in lines 295-305, where this difference could be discussed).

**Response: We have added information to discuss this point in greater detail according to your comment (lines 379-401).**

**Minor comments:**

-L 13-16: I think that the revised description of the objective in the abstract can still be improved. The authors did not only want to identify the consumed pollen but also to quantify their relative importance. I agree with reviewer #1 that there are not measuring preferences explicitly, but at least the relative consumption of the different species was analysed and it would be good to highlight it here.

**Response: We have modified the abstract and introduction section according to your comments (line 14 and 100).**

-L 19-20: Not clear. Did the authors mean that it feeds on more than half of the pollen types offered by the sown plants?

**Response: Yes, we did and have modified the text according to lines 20-21.**

-L 32-33: Agricultural intensification is also very evident in annual crops, perhaps even more than on vineyards and orchards. Maybe changing “especially” for something like “as observed in” can retain the same meaning without emphasizing on the difference between different production systems.

**Response: We have modified the text (lines 36-42).**

-L 38: A better connection with the sentence about the European agri-environment schemes would improve the end of the paragraph, such as “For example, the European…”.

**Response: We have modified the text (lines 44-48).**

-L 40: delete the comma after “defined as habitats”.

**Response: We have deleted the comma (line 47).**

-L 73-74; the expression “are subject to” is not ideal for this sentence. I would say that flowering resources can influence or affect these life-history parameters rather than being subject to them.

**Response: We have modified the text (lines 79-82).**

-L 82-83: this sentence is not clear, particularly the species-rich flowering plants part. Analyses of pollen should be combined with plant inventories or samplings that provide the set of available pollen resources.

**Response: We have modified the text to make the sentence clearer in line with your comment (lines 90-92).**

-L 92-93: I would replace “To achieve this goal, we needed to determine” for a shorter expression such as “Specifically, we were interested in”, or something similar that makes a better connection between the general and specific objectives because those four objectives are specific questions asked by the authors.

**Response: We have modified the text (line 102-103).**

-L 104-105: I understand that this information is necessary for the methods section according to the journal. But I believe that the beginning of the section is not the best option. Perhaps at the end of the paragraph, it would be better?

**Response: The journal requested that we add the sentence (lines 117-118) at the beginning of the paragraph and have followed their instructions. We agree with you that it may be better at the end of the paragraph and have moved the text accordingly (lines 123-125).**

-L 110: can be found is repeated twice in this sentence.

**Response: We have deleted the text (line 123).**

-L111: plant species for what? This is the first time the authors mention the sowing of the flowering plants in methods, so the paragraph should start by saying that in this experimental farm, a set of X plant species were planted, following those criteria.

**Response: We have modified the text according to your comment (lines 126-135).**

-L 119: squares should be replaced by plots or something alike, as the 1x9 m sampling areas are not squares. This is also true for the rest of the paragraph and in other parts of the methods section.

**Response: We have modified the text (lines 136-153).**

-L 122: some plots were not well developed? It should be 120.

**Response: We have modified the text (line 139).**

-L127-130: the writing on this short gives the idea that only those five species were sampled in 2016 and those two in 2017, because of the addition of “the” before the species number. I suggest to change it for something like “Due to their poor development, five species sampled in 2016 (species names) and two species sampled in 2017 (species names) were omitted from the analyses”.

**Response: We have modified the text (lines 144-147).**

-L 191: Please correct, you did not check for differences among years, but among sexes on each year (at least that is shown in results).

**Response: We have modified the text (line 208).**

-L 200: the “also” gives the impression that no differences were found for the percentage of the alimentary canal in the previous sentence, but you did found differences.

**Response: We have modified the text (line 217).**

-L 209: remove the comma after *C. lucasina*.

**Response: We have deleted the comma (line 226).**

-L 224: confusing redaction, rephrase “the pollen types most recorded among the ten most frequently identified”.

**Response: We have modified the text (lines 241-254).**

-L 239-243: what information is provided by these groups? This is not discussed later in the article and it is probably linked with some individuals sharing some pollen types, but if it doesn’t represent something relevant, I would remove this description of the groups from the results.

**Response: Thank you for your comment. These groups provided information on a pattern of relationships between pollen types consumed by *C. lucasina* adults. The absence of a relationship shows that *C. lucasina* adults are opportunistic feeders in our study area. We have thus modified the text (lines 259-271) and have added information to the discussion section (lines 284-434).**

-L 250-253: again, no differences between years were tested, rephrase. Also, the start of the parenthesis is missing and those are several Fisher tests, in the plural.

**Response: We have tested differences between sexes in relation to each fungal spore identified in each year and have modified the text accordingly (lines 276-281).**

-L 288-289: this sentence is not clear.

**Response: We have modified the text (lines 366-368).**

-L 314-324: were these anemophilous plants represented by only a few pollen grains per individual lacewings or by many? In previous articles and my experience, C. sativa could be used as a direct pollen source because many grains can be observed sometimes. But Pinus and other coniferous are sometimes represented by very few grains and this could be a clear indication that lacewings consumed these species on the surface of other plants or with honeydew.

**Response: There was no exclusive zone in the alimentary canal of *C. lucasina* adults filled with anemophilous pollen grains. *P. pinea* type pollen grains were not the principal pollen type in the alimentary canal although there were more than one or two grains per *C. lucasina* adult. We have modified the text to make the sentence clearer (lines 410-419).**

**Reviewer #3:** The authors appear to have carefully addressed all queries and comments raised in review and provision of a marked copy enabled efficient assessment of resubmission.

**Response: Thank you for your comment.**
